# Overcoming substrate limitations for improved production of ethylene in *E. coli*

**DOI:** 10.1186/s13068-015-0413-x

**Published:** 2016-01-04

**Authors:** Sean Lynch, Carrie Eckert, Jianping Yu, Ryan Gill, Pin-Ching Maness

**Affiliations:** Biosciences Center, National Renewable Energy Laboratory, 15013 Denver West Parkway, Golden, CO 80401 USA; Renewable and Sustainable Energy Institute, University of Colorado Boulder, Boulder, CO 80309 USA

**Keywords:** Ethylene, Ethylene-forming enzyme, Arginine, α-ketoglutarate, *E. coli*

## Abstract

**Background:**

Ethylene is an important industrial compound for the production of a wide variety of plastics and chemicals. At present, ethylene production involves steam cracking of a fossil-based feedstock, representing the highest CO_2_-emitting process in the chemical industry. Biological ethylene production can be achieved via expression of a single protein, the ethylene-forming enzyme (EFE), found in some bacteria and fungi; it has the potential to provide a sustainable alternative to steam cracking, provided that significant increases in productivity can be achieved. A key barrier is determining factors that influence the availability of substrates for the EFE reaction in potential microbial hosts. In the presence of O_2_, EFE catalyzes ethylene formation from the substrates α-ketoglutarate (AKG) and arginine. The concentrations of AKG, a key TCA cycle intermediate, and arginine are tightly controlled by an intricate regulatory system that coordinates carbon and nitrogen metabolism. Therefore, reliably predicting which genetic changes will ultimately lead to increased AKG and arginine availability is challenging.

**Results:**

We systematically explored the effects of media composition (rich versus defined), gene copy number, and the addition of exogenous substrates and other metabolites on the formation of ethylene in *Escherichia coli* expressing EFE. Guided by these results, we tested a number of genetic modifications predicted to improve substrate supply and ethylene production, including knockout of competing pathways and overexpression of key enzymes. Several such modifications led to higher AKG levels and higher ethylene productivity, with the best performing strain more than doubling ethylene productivity (from 81 ± 3 to 188 ± 13 nmol/OD_600_/mL).

**Conclusions:**

Both EFE activity and substrate supply can be limiting factors in ethylene production. Targeted modifications in central carbon metabolism, such as overexpression of isocitrate dehydrogenase, and deletion of glutamate synthase or the transcription regulator ArgR, can effectively enhance substrate supply and ethylene productivity. These results not only provide insight into the intricate regulatory network of the TCA cycle, but also guide future pathway and genome-scale engineering efforts to further boost ethylene productivity.

## Background

Ethylene is a versatile hydrocarbon used in the production of a wide range of chemicals including polyethylene (plastic bags and trashcan liners), ethylene oxide (detergents and surfactants), and polystyrene (packaging and insulation) [1]. Consequently, ethylene is one of the most widely used chemicals in the world. Global demand of ethylene is currently met by the steam cracking of fossil fuels, one of the most energy intensive and highest CO_2_ emitting processes in the chemical industry. Approximately two MJ of energy are invested per pound of ethylene produced, which accounts for 1.5 % of the United States’ carbon footprint [1, 2]. Given the ethylene industry’s massive market size and the increasing demand, this footprint will continue to expand without new and innovative methods to produce this multipurpose molecule. Thus a more sustainable route for ethylene production via engineered microbes (bioethylene) would be of great interest to the chemical industry.

In order for a microbial bioethylene platform to be a viable alternative to current ethylene production methods, the rate and yield need to be improved considerably. Hybrid biological–chemical processes for the production of ethylene via dehydration of bio-derived ethanol are efficient in terms of carbon yield. However, insights from microbial ethylene production via genome engineering efforts in model organisms such as *Escherichia coli* will guide engineering of an array of microbes including cyanobacteria for the direct production of ethylene from CO_2_ and sunlight.

**Fig. 1:**
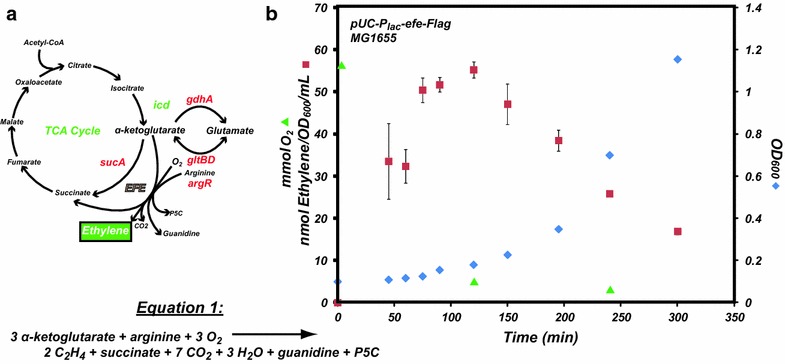
**a** Putative metabolic scheme for ethylene production in *E. coli* via the Ethylene-Forming Enzyme (EFE) and the formation of glutamate from α-ketoglutarate (AKG); genes responsible for the catalytic steps or regulation relevant to this work indicated in *red* (knockout) and *green* (overexpression). **b** Ethylene production, headspace O_2_, and growth over time in LB media for *E. coli* (MG1655) harboring the pUC-Plac-*efe-*Flag plasmid revealing ethylene production to peak at an optical density between 0.2 and 0.3. P5C: L-Δ^1^-pyrroline-5-carboxylate. Gene abbreviations: *icd* (isocitrate dehydrogenase), *gdhA* (glutamate dehydrogenase), *gltBD* (glutamate synthase), *argR* (transcriptional regulator of arginine biosynthesis), *sucA* (2-oxoglutarate decarboxylase)

Biologically, ethylene serves as a plant hormone, modulating growth and development and as a defense response to biotic and abiotic stresses [3, 4]. Due to these roles, a variety of plant-associated pathogens and symbionts have evolved the ability to produce ethylene. While the precise roles ethylene may have in plant disease progression and symbiosis are unclear, evidence suggests that plants infected with certain ethylene-producing bacterial and fungal pathovars such as *Pseudomonas syringae* and *Penicillium digitatum* are indeed compromised [5–7]. These microbes use ethylene-forming enzyme (EFE) to catalyze the formation of ethylene in a single step. The proposed reaction involves both α-ketoglutarate (AKG) and arginine as substrates in the presence of O_2_ [8–11]. In addition to producing ethylene, the proposed reaction also generates succinate, L-Δ^1^-pyrroline-5-carboxylate (P5C), guanidine, and CO_2_ (Eq. 1 and Fig. 1a). While the details of the EFE catalyzed reaction are still being determined [12], ethylene production via a single-enzyme conversion of common metabolites provides a straightforward means to produce bioethylene in engineered hosts such as *E. coli*.

Heterologous expression of EFE and production of ethylene has been demonstrated in a variety of engineered organisms including *E. coli*, *Saccharomyces cerevisiae,* and cyanobacteria [8, 9, 11, 13–19]. Previously published work in *E. coli* yielded productivities approaching 60 mmol/gDCW/hr. Greater productivities have been achieved via EFE expression in native hosts that are less amenable to genetic manipulation (e.g., *P. syringae*), presumably due to an increased availability of substrates in the native hosts [9].

As is often the case with microorganisms engineered for the production of small molecules, limited intracellular availability of substrates, in this instance AKG and arginine, places the most significant limit on ethylene yields. With the exception of *E. coli* and S. *cerevisiae,* there is a noticeable lack of successful strategies in the literature to overcome issues of substrate limitation in engineered hosts. Herein we present a systematic study of the effects of media composition and gene copy number on ethylene production in engineered *E. coli* strains. Working under the hypothesis of substrate availability being the major limiting factor in the production of ethylene, we also explored the effects of reaction substrate and substrate precursor supplementation on ethylene formation. Results from these studies were subsequently utilized to design and test the effects of a series of targeted genetic modifications on the production of ethylene. The parameters for the production of ethylene described here will guide future genome engineering approaches using synthetic biology-enabled tools.

## Results and discussion

### Effects of plasmid copy number and media composition

Measuring ethylene gas requires a closed system, hence preventing a continuous supply of oxygen, a reactant in the proposed EFE reaction (Eq. 1). Moreover, the rate of growth and resultant production of ethylene is also influenced by the media used. Therefore, it was necessary to establish a standard method to measure and compare growth, EFE expression, and headspace content (ethylene, O_2_) of different strains and media conditions. We began by analyzing growth and ethylene production over time in a potentially high-throughput system. The *efe* gene was initially cloned into a high-copy plasmid downstream of an inducible lac promoter (pUC-P_lac_-*efe*-Flag) and transformed into *E. coli* (MG1655) cells. A culture with a starting OD_600_ = 0.1 was split into 1 ml cultures in 2 ml vials sealed with septa for each time point measurement. Previous work has demonstrated that EFE protein accumulated in inclusion bodies in cultures grown at temperatures greater than 30 °C [9], so all of our cultures in this work were grown at 30 °C. At each time point, the headspace was analyzed via gas chromatography (GC) to monitor ethylene (and O_2_), as well as cell growth (OD_600_). This method yielded highly repeatable results at small scale, validating it as a method for comparative studies with a potential for future automation. Our initial results showed that ethylene production peaked near 55 ± 1.9 nmol/OD_600_/mL when cell OD_600_ reached between 0.2 and 0.3 in LB medium (Fig. 1b). Subsequent studies verified this cell density as the point where maximal ethylene production was reached regardless of the media used. Perhaps unsurprisingly, this was also the point of growth at which O_2_ levels were nearly depleted within the sealed culture vial, thus highlighting O_2_ availability as one of the rate-limiting factors for sustained ethylene production. All subsequent measurements of ethylene in this study were performed at a time point where the cell density of the growing culture was between 0.2 and 0.3.

Previous work has shown the importance of promoter strength on the expression of EFE in *E. coli,* with stronger promoters producing elevated levels of ethylene [15]. As such, we chose to replace the relatively weak lac promoter with the *Amaranthus hybridus* chloroplast *psbA* promoter (P_psbA_), known to generate expression levels rivaling those reported with T7 promoters in *E. coli* without requiring IPTG for induction [20]. To determine if high-copy vectors are the best choice for optimal levels of soluble EFE, we also chose to explore the effects of plasmid copy number on the production of ethylene. The P_psbA_-*efe* gene was ultimately cloned into high (pUC-P_psbA_-efe-Flag, ~500 copies per cell)-, medium (pBBR1-P_psbA_-efe-Flag, ~25 copies per cell)-, and low (pRK290-P_psbA_-efe-Flag, 4–7 copies per cell)-copy vectors with the resulting plasmids used to transform *E. coli* (MG1655).

**Fig. 2:**
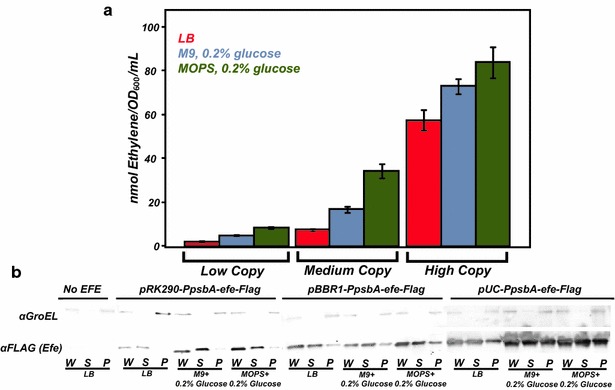
**a** Peak production of ethylene from high-, medium-, and low-copy plasmids in LB media, M9 (0.2 % glucose) and MOPS (0.2 % glucose) minimal media. **b** Western blots of EFE-Flag proteins expressed in *E. coli* (MG1655) from high-, medium-, and low-copy plasmids in different media. In each instance a significant amount of expressed protein remained insoluble. *W* whole cell; *S* soluble; *P* lysed cell pellet

Various culturing media have been used to analyze production of ethylene in *E. coli* including LB and LB medium containing glucose [15]. It has previously been shown that cells grown in M9 minimal medium containing glucose produced elevated levels of AKG, a substrate of the EFE reaction, relative to cultures grown in LB (2.2-fold increase) [21]. We therefore monitored relative production of ethylene in strains carrying each of the three plasmids containing P_psbA_-efe-Flag in LB as well as in M9 and MOPS minimal media containing 0.2 % (w/v) glucose (Fig. 2a).

Results from Fig. 2a clearly show a correlation between plasmid copy number and ethylene productivity with the expression of pUC-P_psbA_-efe-Flag yielding 57, 73, and 81 nmol ethylene/OD_600_/mL during growth in LB, M9 (0.2 % glucose), and MOPS (0.2 % glucose) minimal media, respectively. These results agree with the observed increase of the reaction substrate AKG in cells grown in M9 minimal media [21]. Higher yields of ethylene also corresponded to higher levels of total EFE, in particular soluble EFE, as shown by Western blotting (Fig. 2b), suggesting that concentration of soluble EFE is a rate-limiting factor. Taken together, these observations indicate that both AKG substrate availability and the amount of soluble EFE are limiting factors for ethylene production, hence guiding design principles are important for the construction of future production strains. For example, while it is highly desirable for heterologous genes to be stably integrated onto the chromosome, the levels of ethylene observed from low-copy plasmids are negligible. Therefore, chromosomal integration of the *efe* gene, if necessary, may require further optimization of gene expression, and/or incorporation of multiple copies of *efe* to increase levels of soluble EFE protein.

Although EFE expression driven by stronger promoter from a high-copy number plasmid resulted in the highest ethylene production, a large amount of the total EFE was still insoluble (Fig. 2b). EFE was previously reported to be highly unstable and insoluble at 37 °C while displaying improved stability, solubility, and activity at 23 and 30 °C [9]. Using light- and arabinose-inducible promoters, Digiacomo et al. demonstrated the highest reported levels of ethylene production from EFE in *E. coli* at 37 °C (~25 nmol ethylene/OD_600_/mL) [22]. While below the productivity levels reported here at 30 °C, they do offer a potential strategy to further improve ethylene production at elevated temperatures. Western blots of samples from our analyses indicated a significant amount of the EFE protein remained insoluble under all conditions and expression levels assayed, but the insoluble EFE fraction was most pronounced when expressed from the highest copy number plasmid. Attempts to improve solubility via N- and C-terminal fusions of solubility tags (GFP, Halo, and GST) were ultimately unsuccessful with the C-terminal tags completely disrupting function (data not shown). Fusion of some tags on the N-terminus also seemed to negatively affect EFE function (data not shown). The co-expression of *E. coli* chaperone proteins GroEL and GroES led to slightly higher amounts of EFE protein in the soluble fraction and total amounts of ethylene produced, yet it caused a significant growth defect in cultures co-expressing EFE; these cultures were unable to reach an OD_600_ of 0.25 after 24 h. No further attempt was made to improve EFE solubility in *E. coli* as it will likely require a significant amount of protein engineering and was beyond the scope of the work presented here.

### The effect of nutrient supplementation

**Fig. 3:**
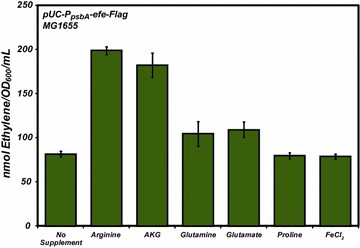
Peak production of ethylene from the wild-type *E. coli* in MOPS minimal media (0.2 % glucose) supplemented with the indicated nutrient at the following concentrations: glutamate (50 mM), glutamine (5 mM), AKG (2 mM), arginine (3 mM), proline (1 mM), and FeCl_2_ (80 µM)

To further test the assumption of substrate limitation as a rate-limiting step, we measured the effects of nutrient supplementation on the production of ethylene (Fig. 3). In addition to supplementation of AKG and arginine, we also measured the effects of glutamine, glutamate, Fe^2+^, and proline addition on ethylene production. With AKG serving as the carbon backbone for both glutamate and glutamine, we reasoned that addition of either to the growth media could improve intracellular AKG by either inducing a signal to limit carbon flux away from AKG or shifting metabolic equilibrium from glutamine or glutamate towards AKG. Recent flux balance analysis also predicted that addition of proline to the growth medium could improve ethylene production in yeast, so it was reasonable to test its effects in *E. coli* [22]. In addition, previous work indicated that addition of Fe^2+^, a reported cofactor necessary for EFE function, improved ethylene production [15]. In each case the concentration of the supplemented nutrient was at least double that of the likely intracellular concentration with the exception of glutamate (~50 %) [23]. The greatest improvement in production was observed via the addition of AKG and arginine to the growth media, with 2 mM AKG and 3 mM arginine more than doubling production compared to cultures grown in standard M9 media (0.2 % glucose): 198 ± 4.7 nmol ethylene/OD_600_/mL and 182 ± 13.9 nmol ethylene/OD_600_/mL, respectively. These data further support the hypothesis that substrate availability is a major limiting factor for ethylene production. The addition of glutamate (50 mM) and glutamine (5 mM) also appeared to improve function, although to lesser degrees. No such improvement was observed in the case of Fe^2+^ (80 µM) and proline (1 mM) addition.

### The effects of gene modifications

**Fig. 4:**
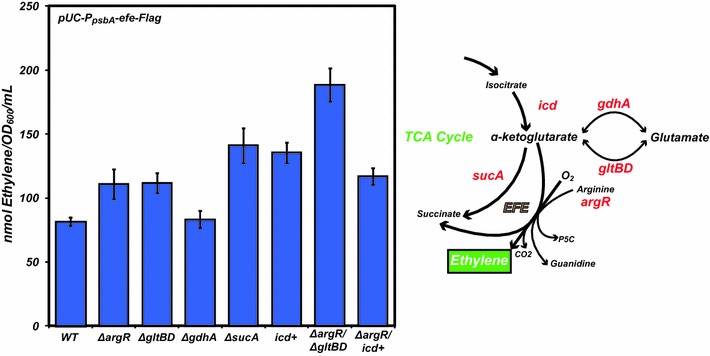
Peak Ethylene production from pUC-P_psbA_-*efe*-Flag in wild-type *E. coli* (MG1655) and each modified strain grown at 30 °C in MOPS minimal media (0.2 % glucose) with the modified genes displayed in *red* on the accompanying pathway figure

Results from the nutrient supplementation above prompted us to design a series of rational genetic modifications predicted to improve AKG or arginine availability within the cell. Because arginine is a co-substrate (in addition to AKG) for the production of ethylene (Eq. 1), we attempted to improve arginine availability via deregulation of arginine biosynthesis. In *E. coli*, arginine biosynthesis is controlled by a regulatory protein encoded by *argR*. Previous work has shown that knockout of *argR* alleviates regulation of arginine biosynthesis resulting in increased arginine availability [24]. As expected, the removal of arginine biosynthesis regulation in the *ΔargRE. coli* strain improved production of ethylene by 36 % compared to the wild-type strain, to 110 ± 11.7 nmol/OD_600_/mL (Fig. 4).

Glutamate dehydrogenase (*gdhA*) catalyzes the NADH-dependent amination of AKG to form glutamate. We reasoned that knockout of *gdhA* could lead to an accumulation of AKG and therefore a boost in the production of ethylene. Similarly, the *E. coli* glutamate synthase catalyzes formation of glutamate from AKG and glutamine. Therefore, knockout of both small and large subunits of the native glutamate synthase (*gltBD)* was also hypothesized to increase AKG accumulation and production of ethylene. The removal of a third AKG-consuming pathway, 2-oxoglutarate dehydrogenase (*sucA*), was also explored. This enzyme catalyzes the formation of succinyl-CoA and CO_2_ from AKG, and deletion of *sucA* results in increased AKG levels in batch culture [25].

In addition to improving substrate availability via removal of competing reactions for its consumption, we also attempted to direct flux towards AKG via overexpression of relevant pathway genes. Many “classic” metabolic engineering methodologies seek to improve the levels of specific metabolites by eliminating the rate-limiting steps involved in its production. Isocitrate dehydrogenase (*icd*) is commonly considered to be the rate-limiting step in the TCA cycle and therefore AKG biosynthesis [26], making it a candidate for overexpression to direct more flux towards AKG.

**Fig. 5:**
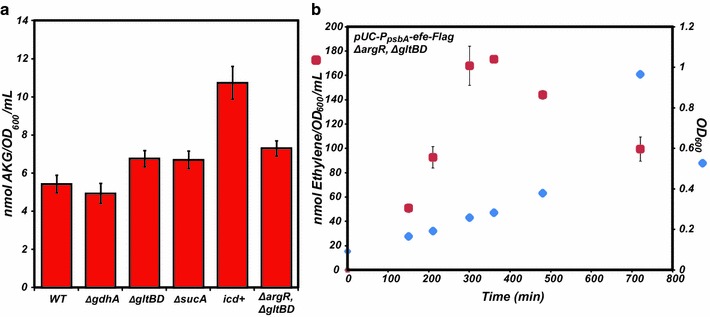
**a** Intracellular AKG levels in wild-type *E. coli* (MG1655) and each modified strain grown at 30 °C in MOPS minimal media (0.2 % glucose). **b** Ethylene production and growth as a function of time for the *ΔargR/ΔgltBD* strain with EFE expressed from the pUC-P_psbA_-*efe*-Flag plasmid

Of the genetic modifications theorized to increase the production of ethylene by increasing AKG availability, only the *ΔgdhA* strain failed to significantly improve production (Fig. 4). The *ΔsucA* and *icd* overexpression strains displayed the highest ethylene measurements, 141 ± 13.6 nmol/OD_600_/mL and 135 ± 7.9 nmol/OD_600_/mL, respectively, in singly modified strains. Of the catalytic steps consuming AKG, the formation of succinyl-CoA is energetically most favored; therefore, it’s sensible to postulate that its deletion would give rise to the largest accumulation of AKG [27]. Similarly, production of ethylene from the *ΔgltBD* strain (111 ± 7.9 nmol/OD_600_/mL) was greater than that from the *ΔgdhA* strain (83 ± 6.6 nmol/OD_600_/mL). This finding is consistent with previous work demonstrating that the *K*_*m*_ for ammonium of the *E. coli* glutamate synthase is significantly lower than that of glutamate dehydrogenase, indicating the preferred pathway for glutamate formation from AKG is through glutamate synthase [28]. To confirm our assumption that increased intracellular AKG is the direct cause for improved ethylene production, each modified strain was assayed for AKG levels in the absence of EFE. Each engineered strain indeed displayed an increased level of AKG on a per cell basis when compared to wild-type, with the *icd*+ strain displaying a nearly twofold improvement in AKG accumulation (Fig. 5a). It should be noted that the *icd*+ strain displayed a significant growth defect, taking 9 h to reach an OD_600_ of 0.22. Interestingly, this growth defect is alleviated by the co-expression of EFE (data not shown). A plausible explanation for this observation may be that significantly increased AKG levels in the *icd*+ strain signal nitrogen deficiency, leading to slowed growth, whereas EFE activity consumes excess AKG and thus rescues growth.

We next attempted to determine which combinatorial mutations might improve production of ethylene. Both AKG and arginine levels within the cell are tightly regulated and vary according to many environmental factors including carbon and nitrogen availability. Many of these properties are interrelated, governed by intricate regulations that are difficult to predict and engineer. For example, recent work has shown that ArgR regulates both arginine biosynthesis and the *gltBD* genes [29]. To explore this dual regulation further, we combined *icd*+ (strain displaying the highest level of AKG) with *ΔargR* and examined the effects on ethylene production. Surprisingly, *icd*+*/ΔargR* had productivity (128 ± 16 nmol/OD600/mL) similar to either singly modified strains *ΔgltBD* (111 ± 7.9 nmol/OD_600_/mL) and *ΔgdhA* (83 ± 6.6 nmol/OD_600_/mL). It is possible that removal of ArgR regulation has the unintended effect of enhancing the transcription of the glutamate synthase genes: increased arginine availability via deregulation of arginine biosynthesis may also increase expression of the glutamate synthase operon, thereby directing flux away from AKG. To further explore this possibility, we chose to create a second double deletion strain in which both *argR* and *gltBD* were removed. The knockout of both *argR* and *gltBD* indeed yielded more than twofold improvement compared to the unmodified strain (188 ± 13.1 nmol/OD600/mL), likely due to the abundance of both AKG and arginine. The production of ethylene for Δ*argR*/Δ*gltBD* was also monitored over time (Fig. 5b). Despite the higher levels of ethylene in Δ*argR*/Δ*gltBD*, this strain also displayed peak production between OD_600_ 0.2 and 0.3 similarly to the single mutants, likely due to the depletion of O_2_ in the head space (Fig. 1b).

The often unpredictable phenotypes of the genomic modifications described above highlight the challenges associated with rational metabolic engineering, especially when genes and pathways involved are tightly coupled to central metabolism. Furthermore, the results of *icd* overexpression suggest the importance of substrate balance (i.e., while AKG is important, arginine plays a significant role as well). Specifically, Fig. 5 shows that *icd* overexpression increases AKG far more than the other perturbations. Yet, despite requiring 3-times more AKG than arginine per putative EFE reaction stoichiometry, its effect on ethylene production is similar to other mutations with lower AKG concentrations. In contrast, the Δ*argR*/Δ*gltBD* double mutant also displays a lower AKG level than *icd*+ but has the highest ethylene productivity. The precise reasons for such phenomena are currently unclear and will require deeper genome-scale investigations before one can more predictably engineer microorganisms for the production of ethylene.

## Conclusion

The engineering of microbes for improved production of ethylene represents a unique challenge; however, many of the risks are offset by less obvious advantages. For example, as a gaseous product, a closed system is required for accurate measurement of production, which in turn limits the availability of molecular oxygen required for EFE function. Yet when compared to other strategies involving the direct production of alcohol- or fatty acid-derived chemicals or fuels, only very small amounts of ethylene accumulates in the media, thus eliminating many of the challenges associated with product toxicity to the host organism and harvesting. Ethylene production therefore has the dual advantages of posing no end product toxicity as well as ease in its harvesting.

Additionally, the extent to which bioethylene will supplant petroleum-derived ethylene will depend largely on the cost and availability of feedstocks. Despite the complexities associated with the rational engineering of central metabolism to improve EFE substrate availability (i.e., central metabolism is highly interconnected) the necessary substrates for production of bioethylene using EFE, namely AKG and arginine, can, depending on the host organism, originate from a diverse set of renewable feedstocks ranging from biomass to CO_2_ and sunlight. For example, production directly from CO_2_ and sunlight in photosynthetic hosts is ideal as they represent the most abundant feedstocks available; however, the engineering of such hosts by high-throughput approaches remains challenging due to a lack of well-established genome engineering methods compared to more tractable hosts such as *E. coli*. It is our aim that some of the insight gained in this work will help guide future engineering efforts in other organisms.

The genetic modifications explored here are certainly not exhaustive; nevertheless they provide an excellent starting position for future genome-scale engineering efforts. In addition to modifying the *E. coli* chromosome, expression of heterologous genes involved in the TCA cycle of other organisms is another avenue that may be explored to bypass innate regulation and improve production. For example, previous work has shown the native *E. coli* citrate synthase, *gltA*, is feedback inhibited by NADH [30]. With NADH being produced in multiple steps of the citric acid cycle, the expression of heterologous citrate synthase proteins not subjecting to regulation by cellular NADH levels could further improve carbon flux towards AKG.

Additional improvements in the production of ethylene using *E. coli* will require both protein engineering to improve EFE activity/solubility and large-scale genome engineering (e.g., Multiplex Automated Genome Engineering (MAGE), Trackable Multiplex Recombination (TRMR)) [31, 32] to further improve EFE substrate availability or co-products recycling. With the vast number of possible mutations that could be introduced to either EFE or the *E. coli* genome and the unpredictable effects of these changes on enzyme function and AKG or arginine availability, improvements in the production of ethylene using evolutionary approaches will rely heavily on the availability of high-throughput screens or selections that tie production of ethylene to increases in cellular fitness. No such tools currently exist; however, given the ubiquitous role ethylene plays in chemical signaling, it is plausible that new high-throughput ethylene-sensing tools can be designed and constructed from the many ethylene-binding proteins found in nature.

## Methods

### General considerations

All plasmid manipulations utilized standard cloning techniques and all constructs were verified by DNA sequencing. Purifications of plasmid DNA, PCR products, and enzyme digestions were performed using kits from Qiagen. Synthetic oligonucleotides were purchased from Eurofins Genomics. All experiments were performed in *E. coli* MG1655 (ATCC).

### Plasmid construction

For P_psbA_-*efe*-Flag plasmid construction, the multiple cloning site (MCS) from pBBR1-MCS1 [33] was flanked with a T7 terminator (*Age*I site) and the *rrnB* T1/T2 terminator (between *Ase*I sites) was either PCR amplified with *Mfe*I ends and ligated into the *Eco*RI site of pRK290 (pRK290MCS1ttori2) or pUC19 (pUC19MCS1ttori1) or digested with BstZ7I and *Nsi*I and ligated to the pBBR1-MCS2 BstZ7I-*Nsi*I backbone fragment (pBBR1MCS2tt). The P_psbA_-*efe*-Flag construct from pJU105 cut with *Sca*I and *Kpn*I (see Ungerer et al.) was inserted into each of the above vectors between the *Eco*RV and *Kpn*I sites of the inserted MCS1tt to ensure that the surrounding DNA sequence is similar for all vectors. To make Plac-*efe*-Flag plasmid, the *efe*-Flag ORF from pJU105 was PCR amplified, digested with *Hind*III and *Kpn*I and subsequently inserted into the pUC-based pGFPuv vector digested with the same enzymes.

The isocitrate dehydrogenase ORF was PCR amplified from the *E. coli* (MG1655) genome, digested with *Hind*III and *Kpn*I and subsequently into the pGFPuv (Clontech) vector cut with the same enzymes. The resulting plasmid was subsequently used as a template for a second PCR reaction to amplify the *icd* ORF now fused to an inducible lac promoter. The resulting PCR product was subsequently digested with *Age*I and *Avr*II and inserted into both a standard pUC19 vector and the above-described pUC-P_psbA_-*efe*-Flag digested with the same enzymes.

### Ethylene measurement

Sequence-verified plasmids were used to transform fresh electrocompetent *E. coli* (MG1655) cells. Cells were plated on LB agar containing the appropriate antibiotic(s) and single colonies were used to inoculate selective liquid media (100 µg/mL carbenicillin, 50 µg/mL chloramphenicol, 50 µg/mL kanamycin for LB and 50 µg/mL carbenicillin, 25 µg/mL chloramphenicol, 25 µg/mL kanamycin for minimal media) and the culture grown to saturation. When appropriate, IPTG was added to a final concentration of 0.5 mM. After 16 h incubation, a 1 mL aliquot of these cultures were harvested via centrifugation and resuspended in fresh selective media and, when appropriate, containing a chosen supplement and diluted to an OD_600_ of 0.1. Aliquots of the diluted culture were grown in 2 mL capped GC vials at 30 °C. A small amount of the headspace was collected at appropriate time points and analyzed using a HP 5980 gas chromatograph under the following conditions: column size, 0.53 mm × 40 m; solid phase, Porapak N column; column temperature, 60 °C; carrier gas, helium; and detector, TCD.

### Knockout construction

The targeted genes were deleted from the chromosome of *E. coli* MG1655 strain using Lambda red recombineering as described previously [34]. Briefly, a linear PCR product containing either a kanamycin or tetracycline resistance marker was amplified from the TKC strain using primers containing 50 bp of homology to the ORF of the targeted gene. A culture of MG1655 carrying the pSIM5 plasmid was grown at 30 °C to an OD_600_ of approximately 0.5 at which time the culture was shifted to 42 °C for 15 min and subsequently made electrocompetent and transformed with the linear PCR product. After at least 2 h of recovery in SOB, cells were plated on selective LB agar. Knockouts were confirmed via PCR.

### Western blotting

For Western blots, cells were resuspended in 1X Bug Buster (Novagen) in 100 mM phosphate buffer, pH 7.0, plus 1X Protease inhibitors (Pierce), and 1 µg/ml Benzoase (Novagen) and incubated at room temperature for 20 min. For soluble versus insoluble fractionation, these whole cell lysates were spun at 14 K rpm, 30 min, 4 °C, and soluble sample was removed. The remaining pellet was washed twice with lysis buffer (spin at 14 K rpm 5 min 4 °C between each) and resuspended in the same volume as that of the removed soluble fraction to represent the insoluble fraction. Protein concentrations were determined by Bradford assay (Sigma), and levels of each sample were adjusted to ∼500 μg/ml. Samples for SDS-PAGE were boiled in 1 × Laemmli sample buffer (Bio-Rad), and 10 μl of each was loaded onto precast TGX Stain-free Any kDa gels (Bio-Rad) and transferred onto a PVDF membrane (Bio-Rad). For Western blotting, the SnapID system (Millipore) was used according to the manufacturer’s instructions. Membranes were blocked in 5 % BSA, 1 × PBST, and primary and secondary antibodies were diluted in 1 % BSA, 1 × PBST. Primary incubation was performed with antibodies against GroEL (Abcam 90522) at 1:5000 as a loading control and the 3XFlag tag on EFE (Clontech Anti-DYKDDDDK) at 1:500 for 1 h. Secondary incubation was performed with Clean blot HRP (Pierce) for 1 h, and Pierce Dura Chemiluminescent reagent was used for signal development. Images were processed using a Cell Biosciences FluoroCam Q Gel imaging system.

### AKG assay

For each measurement, fresh overnight cultures of each strain were used to inoculate 3 screw-cap vials (4 mL) containing 2 mL of MOPS minimal media (0.2 % glucose) to a starting OD_600_ of 0.1. Cultures were grown with shaking at 30 °C to an OD_600_ of ~0.25 at which time 1.5 mL of culture was harvested via centrifugation and resuspended in 100 µL of assay buffer from the Abcam Alpha Ketoglutarate Assay kit (ab83431) and assayed according to the manufacturer’s instructions.

## Abbreviations

AKG: α-ketoglutarate
EFE: ethylene-forming enzyme
P5C: L-Δ^1^-1-pyrroline-5-carboxylate
TCA cycle: tricarboxylic acid cycle
